# Water Fluoridation: A Critical Review of the Physiological Effects of Ingested Fluoride as a Public Health Intervention

**DOI:** 10.1155/2014/293019

**Published:** 2014-02-26

**Authors:** Stephen Peckham, Niyi Awofeso

**Affiliations:** ^1^Centre for Health Services Studies, University of Kent, Canterbury CT2 7NF, UK; ^2^Department of Health Services Research and Policy, London School of Hygiene and Tropical Medicine, London, UK; ^3^e-School of Health and Environmental Studies, Hamdan Bin Mohammed e-University, P.O. Box 71400, Dubai, UAE

## Abstract

Fluorine is the world's 13th most abundant element and constitutes 0.08% of the Earth crust. It has the highest electronegativity of all elements. Fluoride is widely distributed in the environment, occurring in the air, soils, rocks, and water. Although fluoride is used industrially in a fluorine compound, the manufacture of ceramics, pesticides, aerosol propellants, refrigerants, glassware, and Teflon cookware, it is a generally unwanted byproduct of aluminium, fertilizer, and iron ore manufacture. The medicinal use of fluorides for the prevention of dental caries began in January 1945 when community water supplies in Grand Rapids, United States, were fluoridated to a level of 1 ppm as a dental caries prevention measure. However, water fluoridation remains a controversial public health measure. This paper reviews the human health effects of fluoride. The authors conclude that available evidence suggests that fluoride has a potential to cause major adverse human health problems, while having only a modest dental caries prevention effect. As part of efforts to reduce hazardous fluoride ingestion, the practice of artificial water fluoridation should be reconsidered globally, while industrial safety measures need to be tightened in order to reduce unethical discharge of fluoride compounds into the environment. Public health approaches for global dental caries reduction that do not involve systemic ingestion of fluoride are urgently needed.

## 1. Introduction

Community, or artificial, water fluoridation—the addition of a fluoride compound (usually hexafluorosilicic acid) to public drinking water supplies—is a controversial public health intervention; the benefits and harms of which have been debated since its introduction in the USA in the 1950. Discovered by Henri Mossan in 1886, fluorine (F) is a corrosive pale yellow gas. It is highly reactive, participating in reactions with virtually all organic and inorganic substances. Consequently, fluorine is usually found in soil, air, food, and water as fluorides. Fluorine remained a laboratory curiosity until 1940, when nuclear energy requirements stimulated commercial production. In industrial settings, fluorine and its compounds are used in producing uranium, plastics, ceramics, pesticides, and pharmaceuticals. Fluorochlorohydrocarbons are used in refrigeration and aerosol propellant applications [1]. The impact of fluorine on human teeth was recognised in 1909 in Colorado, United States, when two dental surgeons, Frederick McKay and Grant Black, launched an investigation into the causes of mottled enamel (“Colorado brown stain”) in their practice area. Further studies by McKay, Kempf, and Churchill on water samples in areas in Idaho and Arkansas in 1931 confirmed the link between mottled enamel and high water fluoride levels [2]. From 1931, Dr. Trendley Dean, Head of the Dental Hygiene Unit at the National Institute of Health, began investigating the epidemiology of fluorosis. After a decade's study, Dean and his team found that water containing fluoride at a concentration of 1.0 part per million (ppm) appeared to offer some caries protection while minimising the extent of dental fluorosis [3]. However, early studies on the impact of fluoridation on dental caries undertaken by Dean and his colleagues in a Chicago neighbourhood and 12 other cities in four states were qualified; for example,

*“That the inhibitory agent is the fluoride content of the water supply **seems highly probable**. An inspection of the range of dental caries experience associated with the use of domestic water of different fluoride concentration discloses an inverse relation in general between the amount of dental caries and the fluoride concentration of the common water supply. Relatively low dental caries experience rates are found associated with the use of domestic waters whose fluoride concentrations have a range of 1 or more parts per million” *[4]*.*

Further multisite studies commenced in 1945 to determine impacts of fluoridated water on dental caries prevention and health also appeared to demonstrate a positive effect of water fluoridation—with claims of a reduction of dental caries by up to 60% among almost 30,000 schoolchildren in Grand Rapids, MI, USA [5]. However, these findings have been criticised for major methodological flaws, including data cherry-picking and selection bias [6, 7]. Notwithstanding this and before the final results of these studies were known, the US Public Health Service adopted the 1 ppm dose and supported the widespread introduction of community water fluoridation schemes in 1950.

The United States' lead in instituting artificial water fluoridation led to its acceptance by the World Health Organization as an effective oral health intervention. At least 30 nations instituted artificial water fluoridation policies. However, a number of countries including Sweden, The Netherlands, Germany, and Switzerland stopped fluoridating their water supplies due to concerns about safety and effectiveness [8, 9]. Currently, only about 5% of the world's population—350 million people—(including 200 million Americans) consume artificially fluoridated water globally. Only eight countries—Malaysia, Australia, USA, New Zealand, Singapore, and Ireland, more than 50% of the water supply artificially fluoridate. Over the past two decades many communities in Canada, the USA, Australia, and New Zealand have stopped fluoridating their water supplies and in Israel the Minister for Health announced in April 2013 the end of mandatory water fluoridation. However, public health authorities continue to try and develop new community water fluoridation schemes.

The fluoridation debate highlights the dynamics of science and power. To date, the dominant narrative has been that water fluoridation is safe and effective, with advocates claiming strong scientific support and the endorsement of the practice by major dental and public health bodies as evidence of its effectiveness [10]. This is despite key questions about the efficacy and effectiveness of ingested fluoride, concerns about safety, and questions about ethics and legality producing a debate that is a potent mixture of scientific, professional, corporate, and ethical arguments [11–14]. This paper provides a reasoned assessment on the magnitude of the main positive impact of fluoride ingestion on human health (i.e., prevention of dental caries) compared with the established and potential adverse impacts. In particular, it raises questions about what an acceptable safety margin should be for ingested fluoride and questions why normal rules of safety normally applied to assessments of harm and benefit are not applied to water fluoridation. We examine the key arguments and evidence relating to three areas of current debate—efficacy and effectiveness, adverse impacts on health, and ethics. The paper concludes that given the questionable evidence of benefit and increasing evidence of harm the policy of water fluoridation for the prevention of dental caries should be abandoned in favour of more effective interventions combining communitywide and targeted oral health interventions.

## 2. Efficacy and Effectiveness

The only demonstrated positive impact of fluoride on human health is its contribution to prevention of dental caries (infection of teeth enamel). Hydroxyapatite in teeth enamel is made up of calcium, magnesium, and phosphate compounds and is susceptible to decay induced by acid-producing bacteria. Fluoride interacts with hydroxyapatite to form fluoroapatite, which is less susceptible to erosion by acid-producing oral bacteria. About 50% of ingested fluoride is absorbed in the bones and teeth while the rest is excreted in urine. Most of the ingested fluorides reach the teeth via saliva, whose fluoride content varies from less than 0.01 to 0.05 ppm. Fluoride absorption in bones and teeth decreases with increasing age [15]. It is widely accepted that fluoride only helps prevent dental decay by topical means—by direct action on the tooth enamel predominantly after eruption and dental plaque [16, 17]. However, it is important to note that while fluoride contributes to the remineralisation process in the enamel of the tooth surface this is not dependent on fluoride, and that fluoride's anticaries effect is critically dependent on calcium and magnesium content of teeth enamel. Among young individuals with low calcium and magnesium in teeth enamel (usually due to undernutrition), fluoride ingestion and contact with teeth present histologically as hypo-calcification and/or hypoplasia, which may paradoxically make such individuals more vulnerable to dental caries [18, 19]. Fluoride has also been shown to inhibit cariogenic bacteria. This is postulated to occur mainly through inhibition of enzyme-mediated glycolysis in cariogenic microorganisms such as *Streptococcus mutans*. Fluoride is thought to adversely affect polysaccharide metabolism in bacterial cells, reduce the ability of such cells to maintain pH homeostasis, and inhibit encholase as well as other ATPase enzyme systems [20, 21].

Salivary secretions help neutralise caries causing acids and facilitate teeth remineralisation. Individuals with low salivary secretions have higher risk of dental caries [22]. Acid producing normal flora of the oral cavity such as *Lactobacillus acidophilus* thrive and may become cariogenic in the presence of high sugar intake and fermentable carbohydrates on the enamel, as from carbonated drinks. Thus, the multiple pathways to the development of dental caries make it difficult to accurately ascertain the contribution of fluoride ingestion to dental caries prevention. Given that the action of fluoride on dental caries prevention is topical, only topical fluoride products are likely to provide optimal benefits claimed for this chemical.

While early studies of water fluoridation suggested substantial benefits in terms of reduced levels of dental caries, these results have always been contested. Early support was based on an assumed systemic role of fluoride in reducing decay [3, 4]. However, later studies have shown that the differences in fluoride concentration in surface enamel between permanent teeth from areas with no fluoride or low levels and fluoridated areas were minimal and support the fact that effect of fluoride is almost exclusively posteruptive and topical rather than systemic challenging claims made for water fluoridation's efficacy [23–25]. A number of recent studies have questioned whether water fluoridation is effective with studies suggesting no difference in the level of dental caries between children who drink fluoridated water as compared to those who drink nonfluoridated water [26]. Despite this community water fluoridation is endorsed by the World Health Authority, the US Public Health Agency, and most dental and public health organisations as a safe and effective method of reducing dental decay (i.e., caries), a major global public health problem affecting 60–90% of schoolchildren and the vast majority of adults. The World Oral Health Report 2003 concluded that water fluoridation reduces the prevalence of dental caries by about 15% [27]. This lower observation of the contemporary impact of artificial water fluoridation on dental caries was based on a meta-analysis of water fluoridation studies undertaken prior to the late 1990s by the UK NHS Centre for Reviews and Dissemination [28] which found that, for children living in areas where water is artificially fluoridated, the change in the prevalence of dental caries was an average increase of 14.6% in the proportion of children with no dental caries and a decrease of 2.2 dmft in the mean number of decayed, missing, or filled teeth although the studies reported a range of dmft from 0.5 to 4.4, and in terms of the extent of children with caries, the variation was between an increase of 5% and a decrease of 64% [28]. The United States' Centers for Disease Control and Prevention regards water fluoridation as among the top 10 beneficial global health innovations of the 20th century [29]. However, a recent European review recently concluded that water fluoridation is a crude and rather ineffective form of systemic fluoride treatment to prevent dental caries without a detectable threshold for dental and bone damage [30].

In contrast, most studies reporting on the impact of water fluoridation on dental caries prevention appear to suggest that Dean and his colleagues “proved” that water fluoridation reduces dental caries. For example, in a formal statement to commemorate 60 years of artificial water fluoridation, the American Dental Association stated

*“Early studies, such as those conducted in Grand Rapids, showed that water fluoridation reduced the amount of cavities children get in their baby teeth by as much as 60% and reduced tooth decay in permanent adult teeth nearly 35%. Today, studies prove water fluoridation continues to be effective in reducing tooth decay by 20–40%…” *[31]. **

The findings in the York Review [28] that children in fluoridated regions had an average of 14.6% less dental caries is in part reflection of increasing critical assessment of the benefits of community water fluoridation and the impact of improved oral hygiene or other factors. A survey of 55 reputable oral health specialists on the impacts of artificial water fluoridation and other preventive technologies on the decline in dental caries prevalence over the past four decades in most nations revealed that, apart from fluoridated toothpaste, there were conflicting responses on the impact of artificial water fluoridation and other fluoride-based technologies [32]. Studies focused on dental caries trends following cessation of fluoridation have produced contradictory results, in part due to study technique, availability of other fluoride sources, and consumption patterns of cariogenic foods [33, 34].

## 3. Adverse Impacts of Fluoride Ingestion on Human Health

The classification of fluoride as a pollutant rather than as a nutrient or medicine is a useful starting point for analysing the adverse effect of fluoride. No fluoride deficiency disease has ever been documented for humans. Indeed, the basis for setting an "adequate intake" of fluoride rests on the alleged ability of ingested fluoride to prevent tooth decay. However, since it is now known that the effect of fluoride is topical, the notion of an “adequate daily intake” is flawed. One of the key concerns about water fluoridation is the inability to control an individual's dose of ingested fluoride which brings into question the concept of the “optimal dose.” Since the 1980s numerous studies have identified that adults and children are exceeding these agreed limits, contributing to a rapid rise in dental fluorosis—the first sign of fluoride toxicity [35–37]. In 1991, the Centers for Disease Control (CDC) in the USA measured fluoride levels and found that where water is fluoridated between 0.7 and 1.2 ppm overall fluoride, total fluoride intake for adults was between 1.58 and 6.6 mg per day while for children it was between 0.9 and 3.6 mg per day and that there was at least a sixfold variation just from water consumption alone [38]. In their recent review of water fluoridation, the EU (European Union) Scientific Committee on Health and Environmental Risks highlight that young children are likely to exceed the upper tolerable limits for fluoride consumption in areas with water fluoridation greater than 0.8 ppm and using fluoride toothpaste, although the estimates of ingestion are probably underestimated as they are based on ingestion from food and beverages in nonfluoridated areas [30]. Warren et al. have highlighted the complexity of quantifying fluoride intake in areas where there is widespread water fluoridation and increased availability of fluoride-containing products. They argue that “… *it is doubtful that parents or clinicians could adequately track children's fluoride intake and compare it with the recommended level, rendering the concept of an “optimal” or target intake relatively moot*” [26, page 114]. Their conclusion supports earlier research that suggested that the term optimal fluoride intake should be dropped from common usage [39]. As Ismail and Hasson (2008) have argued “*We believe that dentists should dismiss the misconception that there is a balance between dental caries and fluorosis, because patients can accrue the benefits of topical fluorides without developing fluorosis and without systemic intake*” [40, page 1465].

The inability to control individual dose renders the notion of an “optimum concentration” obsolete. In the USA, a study in Iowa found that 90% of 3-month-olds consumed over their recommended upper limits, with some babies ingesting over 6 mg of fluoride daily, above what the Environmental Protection Agency and the WHO say is safe to avoid crippling skeletal fluorosis [41]. Most recently a study in the UK of fluoride levels found in tea concluded that “… fluoride concentrations can exceed the recommended DRI of 4 mg/day…, in certain tea commodities, under the minimal brewing time of 2 min…” [42, page 569]. This study used nonfluoridated water but supports earlier findings by Koblar et al. who report that the adequate intake of fluoride from a 70 kg adult consuming five cups of tea daily ranges from 25 to 210% depending upon tea brand and whether the water is fluoridated [43].

The main source of ingested fluoride to teeth is saliva, whose fluoride concentration is much lower than ingested fluoride. Furthermore, dental caries is essentially the outcome of bacterial infections modulated by physical, biological, environmental, behavioural, and lifestyle-related factors such as high numbers of cariogenic bacteria, inadequate salivary flow, high intake of fermentable carbohydrates, inadequate access to dental services, poor oral hygiene, inappropriate methods of feeding infants, malnutrition (especially calcium and magnesium deficiency), and poverty. Fluoride exposure has a complex relationship in relation to dental caries and may increase dental caries risk in malnourished children due to calcium depletion and enamel hypoplasia, while offering modest caries prevention in otherwise well-nourished children. It has been demonstrated that at low friction loads, enamel hydroxyapatite and fluoroapatite appear to wear in the same way. However, at high friction loads, fluoroapatite enamel flakes and wears catastrophically, leaving severely fractured enamel, whereas hydroxyapetite enamel does not as it is more adaptable to remodelling. This may be due to fluoride's disruption of cycles of demineralisation and mineralisation which take place throughout the lifecycle of teeth enamel [44–46]. The adverse impact of fluoride in producing brittle teeth has been recognised in laboratory animals since 1933, and fluoride-induced brittle teeth were demonstrated to be worse with industrial fluorides such as sodium fluoride compared with naturally occurring calcium fluoride [47].

Sauerheber has analysed the physiologic conditions (such as calcium and pH levels) and systemic effects of ingested fluoride as well as the efficacy of ingested artificially fluoridated water on dental caries prevention [48]. He highlights the important distinction that should be made between naturally occurring fluoride (calcium fluoride CaF_2_) found in water supplies and added fluoride compounds (sodium fluoride NaF and fluorosilicic acid H_2_SiF_6_). His analysis is based on a detailed review of the effect of fluorides on physiological functions and concludes that there are harmful effects from adding artificial fluoride compounds to water supplies. He observes that most analyses of fluoridation rarely focus on detailed physiological analysis but rely on observational epidemiological data to demonstrate effectiveness which are rarely sensitive enough or examine potential issues of harm. One key exception to this was the review by the National Research Council in the USA for the Environmental Protection Agency which took a weight of evidence approach to examining toxicological and physiological effects of fluoride on water [49]. This review identified a number of potential and established adverse effects including cognitive impairment, hypothyroidism, dental and skeletal fluorosis, enzyme and electrolyte derangement, and cancer [49].

In a meta-analysis of 27 mostly China-based studies on fluoride and neurotoxicity, researchers from Harvard School of Public Health and China Medical University in Shenyang found strong indications that fluoride may adversely affect cognitive development in children [50]. All but one study suggested that high fluoride content in water may negatively affect cognitive development. The average loss in intelligence quotient (IQ) was reported as a standardized weighted mean difference of 0.45, which would be approximately equivalent to seven IQ points for commonly used IQ scores with a standard deviation of 15 [50]. While fluoride's effect on IQ in this meta-analysis did not reach statistical significance, the combined effect at population level is remarkable. A particular concern of the NRC committee was the impact of ingested fluoride on the thyroid gland [49]. In a 2005 study, it was found that 47% of children living in a New Delhi neighbourhood with average water fluoride level of 4.37 ppm have evidence of clinical hypothyroidism attributable to fluoride. They found borderline low FT_3_ levels among all children exposed to fluoridated water [51]. The mechanisms through which fluoride exacerbates hypothyroidism include competitive binding with iodine, as well as synthesis obstruction of T_3_ and T_4_. These mechanisms explain the use of fluoride at doses above 5 mg/day in the treatment of hyperthyroidism [52, page 451]. Thus, fluoride-induced hypothyroidism is likely to be more common in iodine-deficient settings. Australian surveys indicate that the general Australian population is mildly deficient in iodine [53] Iodine-deficient children ingesting fluoridated water have been found to demonstrate intellectual deficits even at water fluoride levels of 0.9 ppm [54].

The most obvious and widespread impact of fluoride is dental fluorosis. In some cases—where fluoride levels are very high or where there is prolonged ingestion at 2 ppm or higher, cases of skeletal fluorosis have been reported. Skeletal fluorosis is a chronic metabolic bone disease caused by ingestion or inhalation of large amounts of fluoride. In regions with water fluoride concentrations over 2 ppm, or among workers constantly exposed to fluoride in aluminium or fertilizer industries, skeletal fluorosis is common (>20% prevalence) and manifested as joint pain in both upper and lower limbs, numbing and tingling of the extremities, back pains, and knock-knees. Vertebral osteosclerosis may result in spinal cord compression [55]. In addition, an increase in bone mass due to fluoride ingestion or treatment (for osteoporosis) does not translate into improved bone strength, and high doses of sodium fluoride for osteoporosis treatment may increase the risk of vertebral fractures [56]. Dental fluorosis mirrors skeletal fluorosis. Similar to counterintuitive histological changes in bone, the macroscopic appearance of increasing degrees of dental fluorosis was directly correlated to the degree of subsurface porosity [57]. Despite such histological changes suggesting that tooth decay prevalence may be higher among children with fluorosis, research findings have been mixed [58, 59]. There is no safe limit for fluoride ingestion in relation to dental fluorosis, but fluoridated levels exceeding 0.3 ppm have been associated with teeth mottling and discolouration [30]. Since the initially proposed optimum fluoride intake of 1 mg/day (from one litre of 1 ppm fluoridated water), new sources of fluoride have been introduced through dental care products, processed foods, and commercial beverages. These sources have increased average cumulative fluoride intake to more than 2 mg/day. With these higher levels of fluoride intake, dental fluorosis and other toxic effects noted above have also increased.

Currently, about 41% of children in the United States, where water has been fluoridated at an average level of 1 ppm, have varying degrees of dental fluorosis—levels of over 50% in some fluoridated areas [60]. The National Research Council's report on the health effects of ingested fluoride in the United States, found that “… *the prevalence of dental fluorosis in optimally fluoridated areas (both natural and added) in recent years ranged from 8% to 51%, compared with 3% to 26% in non-fluoridated areas.*” [49, page 37] This implies that while nonwater sources of fluoride are likely to be consumed at the same level in fluoridated and nonfluoridated areas, and while the use of dental supplements is higher in nonfluoridated areas, fluorosis is significantly higher in areas where water is fluoridated. While the only uncontroversial clinical complication of (severe) dental fluorosis is adverse psychological impact on well-being, self-esteem, and negative community perception of affected individuals' oral health [61], established clinical complications of skeletal fluorosis include arthritis, radiculomyelopathy, quadriparesis, and pathological bone fractures [62, 63].

Fluoride is a known enzyme disruptor. For example, fluoride's anticaries effect is derived in part from its ability to derange the enzymes of cariogenic bacteria [20, 21]. Fluoride can interfere by attaching itself to metal ions located at an enzyme's active site or by forming competing hydrogen bonds at the active site which is not exclusively just on the teeth [64]. There are 66 enzymes which are affected by fluoride ingestion, including P450 oxidases, as well the enzyme which facilitates the formation of flexible enamel [65]. A recent study of the effects of inorganic fluoride compounds on human cellular functions revealed that fluoride can interact with a wide range of enzyme-mediated cellular processes and genes modulated by fluoride including those related to the stress response, metabolic enzymes, the cell cycle, cell-cell communications, and signal transduction [66]. Due to high negativity of fluoride, it interacts actively with positively charged ions such as calcium and magnesium. In industrial settings, hydrofluoric acid poisoning is usually treated with intravenous calcium gluconate as such poisoning is associated with acute hypocalcaemia [67]. As with calcium, magnesium plays important roles in optimal bone and teeth formation. By competing with magnesium and calcium in teeth and bones, fluoride deranges the delicate bone formation and bone resorption processes. Such derangements, and consequent intensity of fluoride's adverse effects on bone and teeth, are amplified in malnutrition, calcium deficiency, and magnesium deficiency [68, 69]. Chronic fluoride ingestion is commonly associated with hyperkalaemia and consequent ventricular fibrillation [70].

There have also been a number of studies that link fluoride and cancer. More than 50 population-based studies which have examined the potential link between water fluoride levels and cancer have been reported in the medical literature. Most of these studies have not found a strong link between chronic fluoride ingestion and cancer. In a major review of the topic published in 1987, the International Agency for Research on Cancer labelled fluorides as “… *non-classifiable as to their ability to cause cancer in humans*” and that the studies reviewed “… *have shown no consistent tendency for people living in areas with high concentrations of fluoride in the water to have higher cancer rates than those living in areas with low concentrations*” [71]. However, they concluded that the evidence was inadequate to draw conclusions one way or another and that the evidence linking fluorides with cancer was deemed “inadequate” [71]. The York, NRC and SCHER reviews came to similar conclusions [28, 30, 49] However, population-based-studies strongly suggest that chronic fluoride ingestion is a possible cause of uterine cancer and bladder cancer; there may be a link with osteosarcoma—highlighted as an area where there is evidence of problems requiring further research [30, 72–74].

## 4. Ethical Arguments

Given the uncertainties and debates about effectiveness, efficacy, and the potential for harming health, it is not surprising that community water fluoridation raises important ethical questions. However, these are not restricted to issues of benefit and harm. In addition, community water fluoridation provides policy makers with important questions about medication without consent, the removal of individual choice and whether public water supplies are an appropriate delivery mechanism [75, 76]. Those in favour of water fluoridation have argued that it is ethical as it reduces inequalities in dental health by giving most benefit to children in lower socioeconomic groups [77]. However, the evidence for claiming a reduction in inequalities is generally of poor quality and provides only weak support [28, 76]. Given that more recent studies question whether there is any beneficial impact clearly undermines claims that fluoridation is ethical. Those promoting water fluoridation refer to key legal opinions and ethical assessments often made in the 1960s when there was little questioning of the evidence and a wide acceptance of the benefits of water fluoridation. The Nuffield Council on Bioethics report on Public Health Ethics concluded—water fluoridation presents a number of ethical difficulties due to the poor evidence base, the lack of individual choice, and the fact that it is a universal intervention [75, 76]. As the recent review by SCHER concludes that the balance between harm and benefit in water fluoridation is at best slim and there are more effective interventions for reducing dental decay [30].

One of the early controversies following the completion of the post-1945 Grand Rapids trial of water fluoridation was how fluoride ingested by humans should be classified—a nutrient, medication, or pollutant. Despite numerous studies, the essentiality of fluoride as a trace element or nutrient has not been proven and it is now widely accepted that fluoride is not essential element for human physiology [30, 78]. On 16 March, 1979, the United States Food and Drug Administration deleted Paragraphs 105.3(c) and 105.85(d)(4) of Federal Register which had classified fluorine, among other substances, as essential or probably essential. In an extensive review of fluoride and human health published in 2011, the European Commission's Scientific Committee on Health and Environmental Risks concluded that fluoride is not essential for human growth and development [30]. However, despite widespread recognition of the nonessential nature of fluoride, the Australian National Health and Medical Research Council and the New Zealand Health Ministry currently regard fluoride as a nutrient and have provided nutrient reference values for fluoride and the European Union is currently consulting on whether to set recommended levels for adequate daily intake [79–81].

Although fluoride, used in artificial water fluoridation, is promoted as a medicine for preventing tooth decay, it is not subject to the strict guidelines of medicines statutes in the nations that implement artificial water fluoridation. The practice of water fluoridation is *recommended as a means* of preventing dental caries. Despite this very clear definition of purpose, no fluoridating country defines fluoridation of water supplies as a medicine. As Shaw has recently argued, this classification of fluoridation appears to be based on a legal fiction and argues that artificial water fluoridation is indeed a mass medication and should be subject to strict provisions of Medicines Act in the United Kingdom and similar legislation elsewhere where the practice of artificial water fluoridation occurs [11]. Within the European Union, the only regulation in force for hexafluorosilicic acid—commonly used for community water fluoridation—is as an industrial product.

Arguments in favour of fluoridation as an ethical public intervention rest primarily on the assumption that there are substantive benefits for children's health and that it reduces inequalities. While such claims may have been persuasive several decades ago, this view is clearly now contestable [11, 75, 76, 82]. In an analysis of the evidence and practice of fluoridation in Australia, Awofeso argues that artificial water fluoridation is not just questionable from an ethical perspective but is, in fact, clearly unethical [12].

## 5. Discussion

Fluoride has modest benefit in terms of reduction of dental caries but significant costs in relation to cognitive impairment, hypothyroidism, dental and skeletal fluorosis, enzyme and electrolyte derangement, and uterine cancer. Given that most of the toxic effects of fluoride are due to ingestion, whereas its predominant beneficial effect is obtained via topical application, ingestion or inhalation of fluoride predominantly in any form constitutes an unacceptable risk with virtually no proven benefit. Improvements in occupational health and safety practices and safer disposal of fluoride waste would help to reduce occupational and environmental exposures to fluoride. Artificial or natural fluoridation of water represents a public health hazard—significantly damaging health where fluoride levels are high but are clearly demonstrated as having harmful effects at lower levels found where water has been artificially fluoridated. In addition, ingested water is a very inefficient way of delivering fluoride to teeth given its topical effect but is an important cause of fluoride's adverse effects on human health. Of all sources of fluoride, artificially fluoridated water is the most practical source to eliminate in order to reduce its human hazards at population levels. Indeed, the abundance of fluoride sources ingested by humans, from tea to cereals and condiments [42, 49, 72, 73], suggest that the prime public health priority in relation to fluoride is how to reduce ingestion from multiple sources, rather than adding this abundant and toxic chemical to water or food.

The polarised debate on the role of ingested fluoride in dental health ignores the basic problem that dental caries is essentially the outcome of bacterial infection of teeth enamel. While it might have been excusable in the 1950s to utilise an enzyme poison such as fluoride to undesirably alter dental architecture and to kill cariogenic bacteria, a better understanding of the pathogenesis of dental caries, coupled with development of antibiotics and probiotics with strong anticariogenic effects, diminishes any major future role for fluoride in caries prevention. Newer nonfluoride approaches such as probiotics, Xylitol, and biofilms show increasing promise in caries prevention with a strong safety profile in relation to human health [83, 84]. Since most fluoridation studies have shown that general reductions in dental caries globally have been inequitable despite the introduction of artificial water fluoridation and other fluoride technologies, it is important that caries prevention initiatives are undertaken under the framework of a strong dental health system that integrates nutrition and effective targeted community oral health promotion programmes with accessible dental health services [85].

Although artificial fluoridation of water supplies has been a controversial public health strategy since its introduction, researchers—whom include internationally respected scientists and academics—have consistently found it difficult to publish critical articles of community water fluoridation in scholarly dental and public health journals. Thus, any review of the public health and dental health literature would lead to a bias in favour of water fluoridation. Indeed, the dental public health academic community has sought to brandish opponents to water fluoridation as mad or unscientific [86, 87]. In 2013, residents in Portland, OR, USA, voted for the fourth time to reject the fluoridation of their water supplies. The response by public health authorities was interesting as opponents of water fluoridation were characterised as unscientific and ignoring the needs of children from deprived areas. Almost all articles from peer-reviewed dental journals have been authored by those who support and promote water fluoridation—a situation which continues to the present day—with little critical commentary. Yet there are many papers that raise concerns about the effects of ingested fluoride within the wider scientific literature although there remain few human studies on detrimental health effects and there have been consistent calls for more research in this area [28, 30, 49].

As Wilson and Sheldon have demonstrated dental health policy makers and professionals continue to support water fluoridation and actively promote its use as a safe and effective intervention despite questions about the evidence base [82]. In particular, they note that guidance and policy advice are more likely to draw on sources that do not question the quality or strength of the evidence base. Public health policy makers and professionals continue to promote water fluoridation unquestionably. While there has been some recent reassessment of Dean's optimal level of fluoride concentration, the concept of an optimal dose remains a consistent element of public health policy. For example, in England government, policy is to fluoridate communities at a level of 1 ppm:

*“… the process of adding fluoride to the water supply with a view to reaching a general target concentration of 1 milligram per litre level, or lower if that is not reasonably practicable.” *[88] **

As Verkerk has argued different criteria—which do not follow normal parameters of practice for assessing toxicological affects—are used for fluoride compared to other elements or nutrients [89].

## 6. Conclusion

The enthusiasm with which fluoride was introduced as a public health measure in the 1950s is gradually giving way to a more rational analysis of its benefits and costs as a caries prevention technology. This review argues that the modest benefits of ingested fluoride in caries prevention are thoroughly counterbalanced by its established and potential diverse adverse impacts on human health. Due to the abundance of this chemical, it is little surprising that humans ingest or inhale fluoride from a variety of sources. In the Hippocratic treatise titled *Epidemics*, the ethical principle in relation to controlling disease *Primum non nocere *(“do good or to do no harm”) was emphasised. This principle is, at best, not being fully observed in relation to fluoride-centred dental caries prevention interventions, given the established and potential harms currently attributed to fluoride.

A change in the ideological approach to fluoride use for dental caries prevention is essential in the global public health community. An important change would be for the World Health Organization to repudiate its assertion that fluoride is an essential nutrient or trace element, or that artificial water fluoridation is a useful public health strategy. Resolution 4 of the 2007 World Health Assembly resolution 60.17 urges member states to “consider the development and implementation of fluoridation programmes” [90]. This statement is a reflection of current realities that artificial water fluoridation is not necessarily the most economical, effective, or affordable way to deliver fluoride to teeth in the 21st century [8, 9]. While the statement contrasts with the ringing endorsement provided by the World Health Organization for artificial water fluoridation as recently as 1994, continuing passive support for water fluoridation allows those promoting water fluoridation to use WHO endorsement as an argument for implementing fluoridation programmes [72]. Second, all nutrient values for fluoride need to be withdrawn, not least because it is irrational to have daily nutrient intakes for a hazardous substance whose mode of action is topical on teeth enamel. Third, coordinated global efforts to reduce adverse human health effects on fluoride need to start with ensuring that its introduction into water supplies is prohibited, occupational and industrial fluoride exposures and injuries are reduced to the minimum possible, and natural water systems with high fluoride content are defluoridated prior to being endorsed as “potable.” Finally, given that dental caries is the most common disease globally arising from bacterial infection [91, 92], efforts to develop safe technologies to address the disease deserve high priority. Unfortunately, advocacy for funding to develop nonfluoride approaches for dental caries prevention has so far been compromised by the “religious arguments” between antifluoridationists and profluoridationists.
